# National guidelines for gastric cancer: redundant or needed?

**DOI:** 10.1007/s10120-023-01430-0

**Published:** 2023-10-17

**Authors:** Florian Lordick, Bernhard Wörmann

**Affiliations:** 1 University Cancer Center Leipzig and Department of Medicine II (Oncology, Gastroenterology, Hepatology, and Pulmonology, University of Leipzig Medical Center, Liebigstr. 22, 04103 Leipzig, Germany; 2 Deutsche Gesellschaft für Hämatologie und Medizinische Onkologie, Charité Universitätsmedizin Berlin, Berlin, Germany

**Keywords:** Gastric cancer, Guidelines, Chemotherapy, Authorities

In response to Professor Kodera’s Editorial on the publication of the German, the Austrian and the Swiss Societies for Hematology and Medical Oncology [1] we would like to address a few points. Gastric cancer is a common malignant disease although the age-standardized incidence has been steadily decreasing in Germany, Austria, and Switzerland over the past decades. Annually, approximately 9500 new cases of gastric cancer are diagnosed in men and approximately 6000 new cases in women in Germany. This makes gastric cancer the tenth most common cancer in men, accounting for about 3.5% of all malignant tumor cases, and the ninth most common cancer in women, accounting for about 2.4%. In terms of cancer-related mortality, the relevance of gastric cancer is even higher. Gastric cancer accounts for about 3.5% of all cancer deaths in women and 4.2% in men [2].

The novel edition of the German-Austrian-Swiss gastric cancer guidelines was published a couple of months after the updated international guidelines from the European Society for Medical Oncology (ESMO) [3]. One is tempted to question if separate domestic guidelines are needed and this question was also raised be the Editor-in-chief. We would like to discuss this from the viewpoint of science and from a public policy perspective.

Content wise, the German-Austrian-Swiss guidelines are echoing the overall diagnostic and therapeutic strategies of the European approach, especially when it comes to perioperative chemotherapy for stages IB-III gastric cancer and to sequential palliative chemotherapy lines in stage IV. However, some meaningful differences should be underlined.

Both guidelines deleted their previous optional recommendation for adjuvant radiochemotherapy, given clean resection margins and a D2 lymphadenectomy. In contrast to ESMO, the German-Austrian-Swiss guidelines are not showing adjuvant (postoperative) chemotherapy as an alternative in their graphical illustration and restrict recommendation for neoadjuvant/perioperative chemotherapy (according to the FLOT regimen [fluorouracil, leucovorin, oxaliplatin, docetaxel], if tolerated). This reflects the broad acceptance of the perioperative treatment paradigm in the German speaking parts of Europe, where FLOT for patients with stage IB-III gastric cancer was developed [4].

As a downside of the popularity of FLOT in the German speaking countries, many practicing physicians use the FLOT regimen even in patients with stage IV gastric cancer. For this reason, the German-Austrian-Swiss guidelines emphasize that nowadays only scarce evidence justifies the use of three cytotoxic drugs instead of two in advanced disease stages. The guidelines point out that the additional toxicity of FLOT is not justified in the vast majority of advanced gastric cancer treatment scenarios, and that contemporary studies, including JCOG1013 [5], do not indicate any survival advantage of a triplet versus standard doublet chemotherapy consisting of oxaliplatin plus a fluoropyrimidine.

In view of the recent EU approval of Trastuzumab-Deruxtecan (T-DXd) for patients with advanced HER-2 positive gastric cancer following previous treatment with Trastuzumab, the German-Austrian-Swiss guidelines integrated this novel and clinically effective treatment option into the second-line and beyond algorithm. Of note, the domestic guidelines recommend re-biopsy and assessment of the actual HER2-status before treatment with T-DXd is started, especially when used in second-line where effective alternatives such as Ramucirumab-Paclitaxel are available in case of loss of HER2 positivity. This recommendation also reflects the design of the recently published DESTINY-GC-02 study and is, therefore, science-based [6]. Beyond that, the German guidelines also integrated newly released data and European approval of Pembrolizumab in combination with Trastuzumab and platin-fluoropyrimidine chemotherapy first-line for patients with HER2-positive and PD-L1 combined positive score (CPS) ≥ 1 advanced gastric and esophago-gastric junction adenocarcinoma. Finally, we took position that one of the available immune checkpoint inhibitors (nivolumab or pembrolizumab) should be given in first-line for advanced microsatellite instability (MSI) high gastric cancer. This is not necessarily in line with the approval status of these drugs but reflects consistent and compelling subgroup analyses from several randomized controlled phase III studies and is certainly in the best interest of our patients.

Finally, the German-Austrian-Swiss guidelines provide an outlook on future developments within the treatment landscape, especially regarding targeting of Claudin18.2. As demonstrated in the randomized controlled Spotlight and Glow studies, Zolbetuximab, a Claudin18.2-directed chimeric monoclonal antibody, lead to improved survival outcomes when added to doublet chemotherapy in previously untreated patients with advanced gastric cancer and expression of Claudin18.2 in ≥ 75% of tumor cells [7, 8]. In addition, the authors acknowledge the recently published data from the KEYNOTE-859 study that demonstrates improved overall survival when Pembrolizumab is combined with standard doublet chemotherapy in patients with previously untreated PD-L1-positive advanced gastric cancer [9].

Beyond the scientific content, some other aspects justify the compilation of domestic guidelines complementary to international guidelines. An obvious reason is language: even though the majority of practising oncologists in the German-speaking countries understand English, many prefer to search for medical information in their native language during their busy clinic days. Despite the rapid development of science in oncology, the majority of oncologists in the German-speaking countries continue to treat a broad spectrum of diseases reaching from benign hematology to leukemias, lymphomas and solid tumors. Fast and easy access to medical information is needed more than ever. Onkopedia^®^, a web-based platform provides rapid access to the domestic guidelines (www.onkopedia.de) and is easy to navigate. With an average number of > 50.000 individual or institutional users per month Onkopedia^®^ guidelines are the most popular source of oncology information in the German-speaking countries. Since they are open access, they are also read by the patients themselves. Guidelines in German language reflect our support of transparent patient information and have become an important element of shared decision making.

National guidelines do not only need to illustrate scientific evidence. They also need to mirror clinical routine and access to care in the respective countries. Access to care and reimbursement for cancer drugs differs considerably among European countries and beyond. Without a reflection of actual access to care, recommendations become meaningless for a majority of patients. In Germany, national regulators are legally bound to evaluate novel drugs from a domestic perspective as part of the so-called AMNOG (Gesetz zur Neuordnung des Arzneimittelmarktes) procedure. Their evaluation is the basis for drug pricing and reimbursement. Since 2019, the scientific medical societies are integral part of the early consultation within this Health Technology Assessment (HTA) of new drugs. The experts provide information on the current standard of care, acceptable comparator regimens and clinically relevant subpopulations. Domestic guidelines revealed to be a valuable source of information in this process and have become part of the official documents [10].

The trust in guidelines is key to its success and acceptance in the community. The domestic guideline authors are usually well-known personalities in their countries, nominated by the national scientific societies, and respected by the practising oncologists. The trust in known persons from the own medical community is a key success factor of domestic guidelines.

The national guidelines are complementing the highly successful international ESMO guidelines. ESMO guidelines are an outstanding achievement to leverage evidence-based oncology in Europe and beyond. They build on scientific evidence and expert-consensus. Therefore, they provide levels of evidence and grades of recommendation. For quality assurance, they are anonymously peer-reviewed before published. Additionally, expert groups within ESMO rate interventions according to the Magnitude of Clinical Benefit Scale (MCBS) and biomarkers according to the ESMO Scale for Actionable Targets (ESCAT). No doubt that the ESMO guidelines are the king, while the domestic guidelines are the queen. However, sometimes the queen seems to be closer to the people’s hearts and needs.
